# Ancient Skeletal Evidence for Leprosy in India (2000 B.C.)

**DOI:** 10.1371/journal.pone.0005669

**Published:** 2009-05-27

**Authors:** Gwen Robbins, V. Mushrif Tripathy, V. N. Misra, R. K. Mohanty, V. S. Shinde, Kelsey M. Gray, Malcolm D. Schug

**Affiliations:** 1 Department of Anthropology, Appalachian State University, Boone, North Carolina, United States of America; 2 Department of Anthropology, Deccan College, Deemed University, Pune, India; 3 Indian Society for Prehistoric and Quaternary Studies, Deccan College, Deemed University, Pune, India; 4 Department of Biology, University of North Carolina Greensboro, Greensboro, North Carolina, United States of America; University of Cambridge, United Kingdom

## Abstract

**Background:**

Leprosy is a chronic infectious disease caused by *Mycobacterium leprae* that affects almost 250,000 people worldwide. The timing of first infection, geographic origin, and pattern of transmission of the disease are still under investigation. Comparative genomics research has suggested *M. leprae* evolved either in East Africa or South Asia during the Late Pleistocene before spreading to Europe and the rest of the World. The earliest widely accepted evidence for leprosy is in Asian texts dated to 600 B.C.

**Methodology/Principal Findings:**

We report an analysis of pathological conditions in skeletal remains from the second millennium B.C. in India. A middle aged adult male skeleton demonstrates pathological changes in the rhinomaxillary region, degenerative joint disease, infectious involvement of the tibia (periostitis), and injury to the peripheral skeleton. The presence and patterning of lesions was subject to a process of differential diagnosis for leprosy including treponemal disease, leishmaniasis, tuberculosis, osteomyelitis, and non-specific infection.

**Conclusions/Significance:**

Results indicate that lepromatous leprosy was present in India by 2000 B.C. This evidence represents the oldest documented skeletal evidence for the disease. Our results indicate that Vedic burial traditions in cases of leprosy were present in northwest India prior to the first millennium B.C. Our results also support translations of early Vedic scriptures as the first textual reference to leprosy. The presence of leprosy in skeletal material dated to the post-urban phase of the Indus Age suggests that if *M. leprae* evolved in Africa, the disease migrated to India before the Late Holocene, possibly during the third millennium B.C. at a time when there was substantial interaction among the Indus Civilization, Mesopotamia, and Egypt. This evidence should be impetus to look for additional skeletal and molecular evidence of leprosy in India and Africa to confirm the African origin of the disease.

## Introduction

Leprosy is a debilitating but treatable disease caused by infection with *Mycobacterium leprae*. Although popular conceptions of leprosy are focused primarily on images from Biblical or Medieval times, one quarter of a million people worldwide were still suffering from the disease in 2007—primarily in rural areas of Bangladesh, Brazil, China, Democratic Republic of Congo, Cote D'Ivoire, Ethiopia, India, Indonesia, Mozambique, Myanmar, Nepal, Nigeria, Philippines and Sudan [1]. The history of leprosy is “interwoven with civilization itself” [2]. An understanding of the origin and transmission routes of this disease can potentially lead to new insights about the evolution of infectious diseases and eradication efforts. However, the disease is difficult to culture *in vitro* and much about leprosy is still poorly understood, including the origin, initial transmission routes, and timing for the spread of the disease in the Old World.

The earliest textual references to leprosy are found in proto-historic texts, including the Egyptian Ebers papyrus dated to 1550 B.C. [3]. It has been suggested that there are references to the disease in Sanskrit hymns of the *Atharva Veda* composed before the first millennium B.C. [4] and the Old and New Testaments of the Bible [5], [6]. However, this evidence is controversial [3], [5], [6] and the earliest widely accepted references to the disease are from much later sources: South Asian texts *Sushruta Samhita* and Kautilya's *Arthashastra* dated to the 6^th^ century B.C. [4], [7], 4^th^ century accounts of the Greek author Nanzianos [8], a 3^rd^ century Chinese text *Shuihudi Qin Jia* [9], and 1rst century A.D. Roman accounts of Celsus and Pliny the Elder [5], [6], [10].

Historians of the disease have maintained that leprosy originated in the Indian subcontinent and spread to Europe after the fourth century B.C. [5], [6], [11], [12], [13] but the disease did not become a serious public health problem in Europe until the Middle Ages [10]. Asylums were established by the 7^th^ century in France [14] and skeletal evidence for the disease is well documented for Medieval European skeletal collections from the United Kingdom [10], [15], [16], [17], Denmark [18], Italy [19], Czech Republic [14], and Hungary [20], [21].

Although urbanization has traditionally been considered requisite for the spread of the disease in the Old World [8], genomics research has indicated a Late Pleistocene model for origin and transmission out of Africa [12]. Archaeological evidence for the disease in Africa and Asia in prehistory has also provided indications that the disease has ancient roots. Skeletal evidence of leprosy has been documented in the 2^nd^ century B.C. in Roman period Egypt [22], [23], the 1^rst^ millennium B.C. in Uzbekistan [24], Nubia in the 5^th^ century B.C. [25], and Thailand *circa* 300 B.C. [26]. The earliest documented cases in West Asia (Israel) are from the 1^rst^ century A.D. [27], [28], [29]. Previously there was no skeletal evidence for the disease in South Asia.

**Figure 1:**
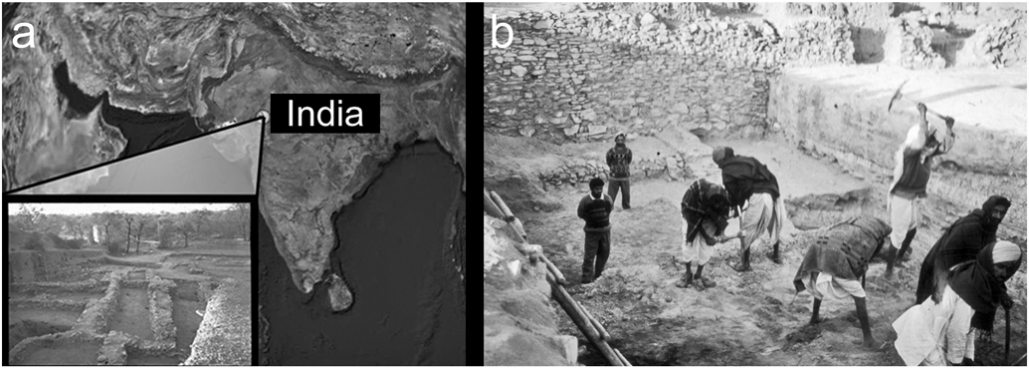
The excavation site in Balathal. A) A map of India showing the location of Balathal and a view of the lower town. B) Photograph of the excavations within the stone enclosure where skeleton 1997-1 was located. This individual was interred in the Chalcolithic deposit (layer 7) of stratified layers of burned cow dung. Associated radiocarbon dates indicate an antiquity of cal B.C. 2000.

We report here on skeletal evidence for leprosy from 2000 B.C. at the site of Balathal (24°43′N 73°59′E), located 40 km northeast of Udaipur in the contemporary state of Rajasthan, India (Figure 1a). There are two phases of occupation represented at Balathal, a small occupation in the Early Historic period (cal. B.C. 760 - A.D. 380) and a large Chalcolithic settlement (cal. B.C. 3700–1820) [30]. The Chalcolithic people of Balathal lived in stone or mud-brick houses, made wheel thrown pottery, copper implements, and practiced dry field agriculture focused on barley (*Hordeum vulgare*) and wheat (*Triticum spp*.). The Chalcolithic deposit demonstrates evidence of Harappan influences in the classical tan ware ceramics, which resemble Harappan red ware in manufacture, fabric, firing, and vessel forms [31]. Copper objects include razor blades, knives, chisels, arrow heads, spearheads, and axes. Two burials were recovered from the 1994–1997 excavations of the Chalcolithic deposit—individuals 1997-1 and 1997-2. Three additional burials were recovered in the 1999–2002 excavations of the Early Historic period—individuals 1999-1, 1999-2, and 1999-3 [32].

**Figure 2:**
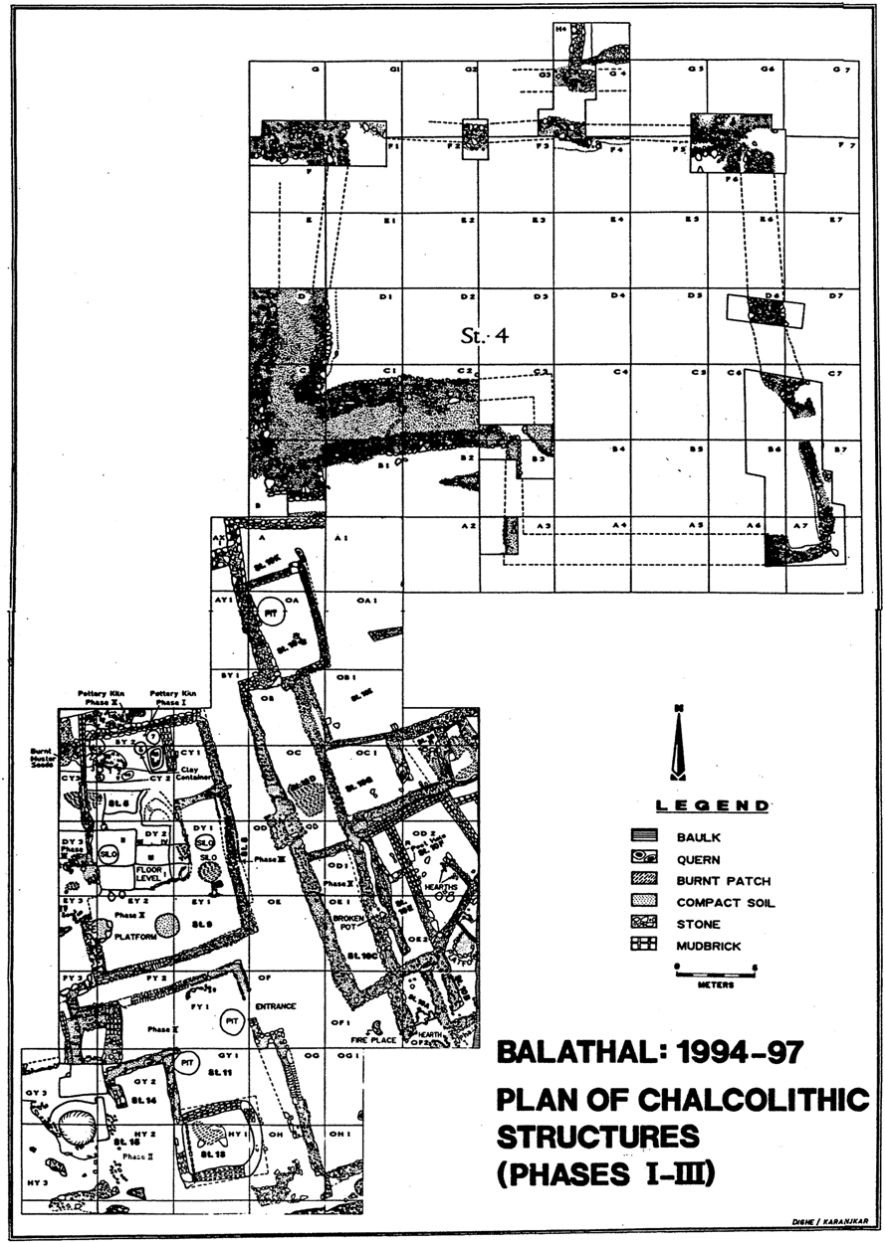
Plan view of the Chalcolithic occupation at the site of Balathal. Balathal Phases I–III Chalcolithic structures uncovered during the 1994–1997 excavation seasons. The skeleton was uncovered in layer 7 of quadrant E3 and the radiocarbon date of 2000 B.C. was obtained in layer 7 of quadrant F4, both of which are within the stone enclosure. The Early Historic phase is not represented here as that portion of the site was excavated in 1999–2002.

This paper concerns individual 1997-1 who was buried inside a stone enclosure at Balathal. The stone enclosure was a Chalcolithic construction overlain by an undisturbed layer (layer 5) of sterile, white ashy soil 20–30 cm in thickness. This sterile layer separated Chalcolithic from Early Historic deposits over the entirety of the mound. This layer accumulated over a span of 1000 years from 1800–800 B.C. during a time of increasingly aridity in western India [33], [34], [35], [36], [37]. The enclosure (500 m^2^) was built at the eastern periphery of the settlement. The walls measure 27×37 m in length and it was built around a foundation 70 cm thick, constructed of mixed clay, silt, brickbats and bricks. The walls of the stone structure are thickest at the base (6.5 m thick) and taper (to 4 m thickness) toward the top of the construction, which along with the platform foundation, is a construction style that resembles Indus citadel construction at Kuntasi and Rojdi in Saurashtra, Gujarat [31]. A radiocarbon date from Layer 13 in Trench E4 (Figure 2) dates the earliest deposits of ash to 3350 B.C. (cal. B.C. 3620–3100). The presence of monumental architecture and new ceramic styles at Balathal from 2400–1700 B.C. has been interpreted as evidence for contact with the Indus civilization during this phase [33].

Radiocarbon dates of the stratified layers in the excavated site provide definitive evidence that the skeleton was buried between 2500–2000 B.C. Inside the stone enclosure there are stratified layers of vitrified ash from burned cow dung that appears to have been thrown into this space from the top of the stone wall (Figure 1b). Individual 1997-1 was interred in a tightly flexed posture, on its left side within undisturbed stratified layers of the burned cow dung (at a depth of 2.66 m, in layer 7 of the Northeast Quadrant of trench E3). There are 45 radiocarbon dates for the entire site of Balathal, 30 from the Chalcolithic layers, perhaps the most complete assessment of radiocarbon chronology for any South Asian site. All of the dates from within the stone enclosure were from the Chalcolithic period [30], which spanned the calibrated date range of 3700–1800 B.C. according to 25 radiocarbon dates [30], [33]. Two radiocarbon dates were obtained from charcoal recovered from Layer 7 in the stone enclosure. A date of 2000 B.C. (cal. B.C. 2200–1980) was obtained from trench F4. A date of 2550 B.C. (cal. B.C. 2830–2310) was obtained from Layer 7 in trench D4. Thus the skeleton was buried sometime between 2500–2000 B.C.

## Methods

Individual 1997-1 was inventoried and described [32] using standard macroscopic techniques in bioarchaeology [38]. This individual is estimated to have been a male based on pelvic architecture [39], a determination supported by skeletal size and robusticity. The innominates are fragmentary but the right and left auricular surfaces, the left sciatic notch, and the right pubis are preserved. There is no pre-auricular sulcus and the sciatic notch is narrow. The right pubic bone has a narrow sub-pubic angle and a rhomboid shape, indicating that this individual was male. Age was estimated based on the pubic symphysis [40] and dental attrition [41]. The form of the pubic symphysis indicates that this individual was 37+/−5 years old when he died. This individual suffered from antemortem tooth loss, which combined with other oral pathologies (described below) could certainly influence the amount of wear on the remaining teeth [32]. The technique yielded an age estimate of 35+/−10 years, which is consistent with the estimate from the pelvis. The length of the humerus provided an estimate for stature of 1.78+/−0.04 meters [42]. Differential diagnosis was undertaken through a comparison of the presence and patterning of lesions in the skeleton with expectations from the paleopathology literature.

## Results

**Figure 3:**
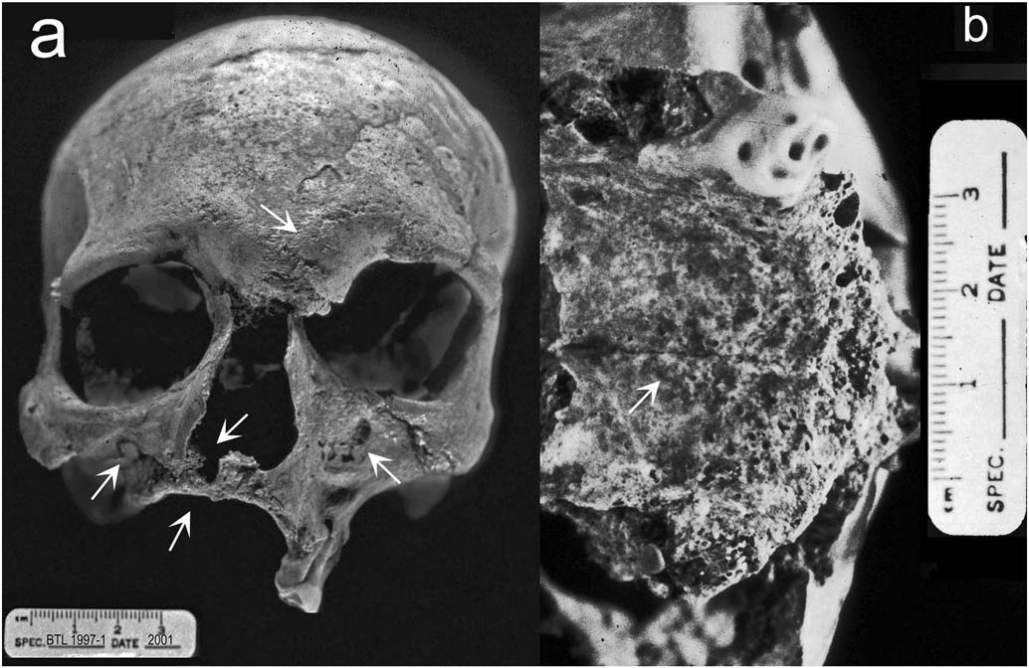
The cranium of individual 1997-1. A) Anterior view demonstrates bilateral erosive lesions at the supraorbital region and glabella, erosion/remodeling of the margin of the nasal aperture, including the anterior nasal spine, bilateral necrosis of the infraorbital region of the maxilla, and resorption of the alveolar region of the maxilla with associated antemortem tooth loss. B) Inferior view of the maxilla demonstrates pathological changes to the palatine process including pitting near the midline and in the alveolar region.

This individual was preserved with a fairly complete skull but the postcranial skeleton is incomplete and fragmentary [32]. Evidence for bone pathology on the facial skeleton includes erosion/remodeling of the lateral and inferior margins of the nasal aperture, complete atrophy of the anterior nasal spine, bilateral osteolytic lesions at the infraorbital region of the maxilla, evidence for infection in macroporosity of the supraorbital region at glabella, and resorption of the anterior alveolar region of the maxilla (Figure 3a). The palatine process of the maxilla also demonstrates pathological changes including pitting near the midline and in the alveolar region indicating superficial inflammation affected regions that had not already resorbed (Figure 3b).

**Figure 4:**
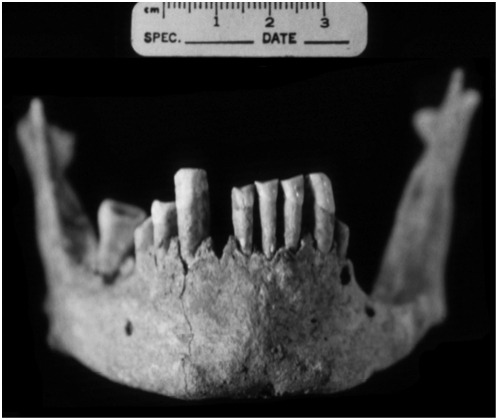
Anterior view of the mandible from individual 1997-1. The mandible demonstrates root exposure, alveolar resorption, antemortem tooth loss, and a small apical abscess at the left third premolar.

Antemortem tooth loss affected the majority of the maxillary teeth, with only the left first molar and fourth premolar remaining *in situ*. There are two large periapical abscesses on either side of this molar but there is no other evidence of pulp chamber exposure or abscessing. Slight traces of the alveoli remain for the right canine, third premolar, second and third molars and the right second molar is present as an isolated tooth. The molar roots demonstrate a thickening of the apices indicative of hypercementosis. Antemortem tooth loss and alveolar resorption has also affected the mandible (Figure 4) but eight mandibular teeth remain *in situ*—right and left central and lateral incisors, canines, right third premolar, and the right third molar. Alveolar resorption and passive eruption in the anterior mandible has exposed an average of 7 mm of root surface in the incisors and canines. Resorption in the left posterior mandible has obliterated the alveoli and only a thin segment of the mandibular corpus remains.

**Figure 5:**
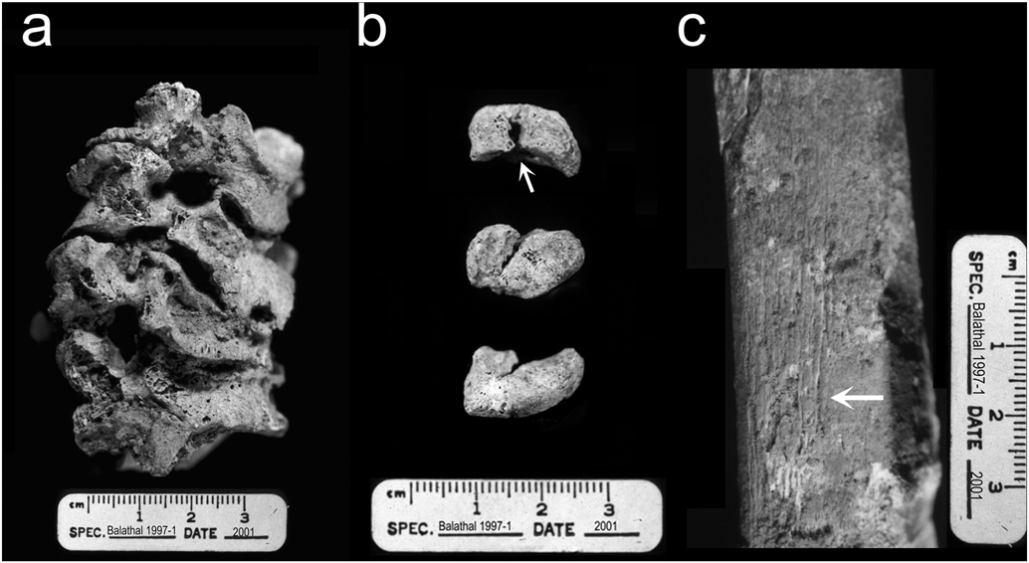
Elements demonstrating pathological conditions in the postcranial skeleton of individual 1997-1. A) Left lateral view of the cervical vertebrae (C3–C7) demonstrates degenerative changes including ventral wedging, osteophytosis, and ankylosis. B) Three views (from the radius, from the triquetral, and the palmar-distal surface) of the left pisiform demonstrating a fracture on the articular surface for the triquetral. C) Lateral view of the tibia midshaft. Arrow points to periostitis on the compact bone surface.

In the postcranial skeleton, there is evidence for extensive degenerative disease with marginal osteophytes affecting most of the joint surfaces present, including the right and left glenoid fossae of the scapulae, left humerus (proximal epiphysis: head and trochanters), right and left ulnae (lunar and radial notches), left radius (distal epiphysis), the vertebral ends of the right and left ribs, left innominate (around the perimeter of the acetabulum), the right and left femoral heads, and the proximal end of the left tibia (lateral condyle). The fourth through the seventh cervical vertebrae had severe degenerative changes including ventral wedging, osteophytic lipping on the margins of the centra and on the superior and inferior articular surfaces, and vertebral ankylosis, or fusion of the cervical vertebrae (Figure 5a). Similar changes were noted on the lumbar vertebrae (L3–L5). The left pisiform is present and there is a fracture on the articular facet for the triquetral (Figure 5b). The proximal half of the left and right tibiae are present and the compact bone surface on the right is irregular and evidence for infection (periostitis) is present (Figure 5c)

The distal end of the right radius, ulna, and left triquetral are present and show no evidence of pathology. Many of the elements in the distal ends of the legs are missing—the distal tibiae, fibulae, and many of the foot bones are missing or damaged postmortem. More specifically, the left medial and intermediate cuneiforms and cuboid are present but damaged postmortem. All five right metatarsals are present though they have also suffered destruction of the articular ends. Seven pedal phalangeal fragments are also present but demonstrate no pathological modification.

The distribution of skeletal pathologies is key to a diagnosis of leprosy [6]. We expect leprosy to include changes to the skull and the postcranial skeleton: “cortical inflammatory changes of the palatine process of the maxilla, diaphyseal cortical surface, and intra-articular cortical surface” [43]. The principle change to the skull with leprosy is rhinomaxillary syndrome, which involves loss of bone around the pyriform aperture, destruction of the nasal spine, and loss of bone at the anterior alveolar process [5], [6], [18], [44], [45]. Leprosy is also associated with pathological remodeling of the facial skeleton at the nasal conchae, infraorbital, and palatal regions, including pitting of the cortical surface indicating increased osteoclast activity and/or bone necrosis [46]. In the Balathal skeleton, we have clear evidence of rhinomaxillary syndrome and bilateral expression of infection in the splanchnocranium. These changes are specifically associated with lepromatous leprosy. Unilateral facial lesions are more common in the tuberculoid form of leprosy [43]. There is evidence of a slight amount of pitting at the midline on the palatine process of the maxilla but no evidence of perforation, although the dorsal part is broken. Unfortunately, the nasal conchae are missing postmortem.

Postcranial manifestations of leprosy take two forms: direct bacterial invasion by contact with infected elements and injury to appendages related to leprous autonomic neuropathy [46]. The former can be manifest in non-specific inflammatory changes at multiple sites while the latter can be manifest in evidence for traumatic injury in wrist, hand, ankle, and foot bones. Injuries to extremities are not direct evidence for leprosy but they do corroborate the other evidence as they can be associated with the neuropathy accompany infection with leprosy [6], [47], [48]. For this individual from Balathal, postcranial pathologies include degenerative changes in the spine and diarthrodial joints, infectious involvement of the lower leg, and evidence for injury to the left wrist. Evidence of direct involvement of the hand and foot bones is unavailable although absence of many hand and foot bones could be explained by bone absorption, which would leave the bones more fragile and likely to degrade after burial.

We argue here that these changes are strong evidence for the manifestations of leprosy in 1997-1. Other potential diagnoses include treponemal infection, leishmaniasis, sinus and oral infections, tuberculosis, osteomyelitis and non-specific infection in the post-crania. In cases of treponemal disease, remodeling of the nasal aperture, including loss of the nasal spine, can occur [49]. However, this individual demonstrates no evidence of other diagnostic criteria for adult treponemal infection including caries sicca, widespread periostitis in the axial and appendicular skeleton, thick or irregular long bones, or saber tibiae [5], [50]. Periodontal disease and/or caries can lead to antemortem tooth loss and destruction of the alveolar bone in the maxilla and the mandible [51]. Oral infections and rhinomaxillary sinusitis can cause inflammatory changes to the rhinomaxillary region [52]. Leishmaniasis can also cause destructive lesions of the face, particularly periosteal rections around the nasal spine [50]. However, antemortem tooth loss, oral infections, and leishmaniasis are not known to cause destruction of the pyriform aperture and nasal spine, which are diagnostic criteria for leprosy and are present in individual 1997-1.

This individual does not demonstrate some of the classic manifestations of tuberculosis, a chronic infection by a related group of related *Mycobacteria*, often transmitted through the respiratory system or the digestive tract [50]. Individual 1997-1 demonstrates vertebral ankylosis, which can be associated with spinal tuberculosis in the adult skeleton. However, this individual from Balathal does not demonstrate other pathognomic changes of chronic tuberculosis such as osteoporotic changes in the thoracic and lumbar vertebral centra or kyphosis. In cases of tuberculosis, ankylosis can also affect the knees and hip as a result of septic arthritis [53]. The pathological changes to the joint surfaces in individual 1997-1 are confined to marginal osteophytes that are typical of degenerative joint disease and/or advanced age.

There is no evidence in individual 1997-1 for involucrae, or sequestering of necrotic bone lesions typical of osteomyelitis nor for infectious involvement of the ribs or spine [5], [6], [50]. In the postcranial skeleton, non-leprous osteomyelitis is a product of haematogenous spread of bacteria (usually *Staphylococcus* or *Streptococcus*) often as a result of injury. This condition is characterized by intermedullary abscess and cloaca formation in the spine, ribs, femur, tibia [43], [50]. Individual 1997-1 does demonstrate periostitis in the tibia that could result from leprosy or some other, non-specific infection. Given the patterning of lesions, the absence of key diagnostic criteria for treponemal infection, tuberculosis, and osteomyelitis, it is argued here that this skeleton represents the oldest example of lepromatous leprosy in the world.

## Discussion

While it has long been thought that leprosy originated in the Old World [5], less is known about the origin and prehistoric transmission routes for leprosy than other related infectious diseases [53]. Our evidence supports Sanskrit translations of the *Atharva Veda* that reference leprosy [4] and supports the suggestion that this ancient text is the earliest historical reference to the disease, its pathogenesis and treatment.

“Born by night art thou, O plant, dark, black, sable. Do thou, that art rich in colour, stain this leprosy, and the grey spots! … The leprosy which has originated in the bones, and that which has originated in the body and upon the skin, the white mark begotten of corruption, I have destroyed with my charm.” (pg. 19)

As the Sanskrit word *kushtha* referred to a plant used to treat leprosy and tuberculosis (*rajayaksma*) [7], the *Atharva Veda* is also the earliest text to infer a connection between the two conditions, at least in terms of treatment. It is not common to find adult burials after 2000 B.C. In contrast, infants and children under 5 years of age are common in peninsular sites. These features of second millennium burial practice are suggestive of Vedic tradition. Given this, it is interesting to note that it is customary in Vedic tradition in parts of India to bury lepers alive [54], [55] rather than cremate their bodies, which as diseased, are not considered an appropriate sacrifice to Hindu Gods [54]. The biological evidence presented here indicates that similar mortuary behavior for people with leprosy was present at a rural Chalcolithic village in northwest India by the beginning of the second millennium B.C.

As far as we are aware, this burial from Balathal is also the earliest example of an individual buried in vitrified ash from cow dung prior to the ash circle burials of the Southern Neolithic. Large stratified deposits of ash are common in the Southern Neolithic ash mounds of the South Deccan and Northern Dharwar region of the contemporary state of Karnataka. Over 100 ash mound sites have been identified as belonging to the Southern Neolithic period but they are not very well understood [56]. The most common interpretation of the ash mounds based on excavations at Budihal and Utner is that they are remains of cattle pens or efforts to rid settlements of cow dung [56]. One alternative hypothesis is that they represent remains from funerary practices [57]. Some of these ash mounds are associated with megalithic monuments, thousands of which cover the landscape of peninsular India. These stone circle burials are occasionally found near ash circle burials but these are a less common tradition in the southern Iron-Age (800–500 BC). The occasional presence of ash circle burials in South India has been interpreted as evidence for integration of burial traditions from the Chalcolithic and Iron Age [57]. The evidence from Chalcolithic Balathal also serves as a bridge between northwestern Chalcolithic traditions and the burial practices of Southern India in the first millennium B.C.

Evidence for leprosy in India at 2000 B.C. can be used to address hypotheses about prehistoric transmission models for the disease. Although leprosy is often considered to have a recent origin [5], [6], [44], analysis of rare single nucleotide polymorphisms in contemporary samples of *M. leprae* from worldwide geographic regions [12] identified two strains of leprosy segregating in Asia (predominantly Type I) and east Africa (Type II). Because of the low frequency of the Type II strain in Asia, and its high frequency in East Africa, one scenario for leprosy's origin is that Type II evolved first in East Africa (before 40,000 B.C.) and was later transmitted to Asia (evolving into Type I) and Europe (evolving into Type III), which is also common in West Africa and the Americas [12].

Alternatively, the Type II strain may have evolved from the Type I strain in Asia much more recently and was then transmitted out of Asia, into Africa and Europe [8]. Small sample sizes and potentially biased demographic sampling of *M. leprae* from contemporary populations in the comparative genomics study could explain the absence of the Type II strain in South Asia (n = 4). Sampling issues or fixation of the Type II strain in East Africa (n = 2), combined with contemporary eradication efforts in India may have lead to an underestimate of the putative ancestral Type II strain's historical prevalence in India, and the derived Type I strain's historical prevalence in East Africa.

The Late Holocene transmission scenario is more compatible with the natural history of *M. leprae*, which thrives on human contact and may have spread to East Africa during the development of urban life and large inter-continental trade networks during the height of the Indus civilization and the “Middle Asian Interaction Sphere” [58]. The “Middle Asian Interaction Sphere” is a term used to describe political and economic contacts between South and West Asian Bronze Age peoples in the third millennium B.C. There are four core areas involved—Meluhha in the Indus Valley, Turan in Central Asia, Mesopotamia in the Fertile Crescent, and Magan on the Arabian Peninsula. The evidence for inter-regional interaction includes textual sources from Mesopotamia indicating trade relationships with Meluhha from the Early Dynastic Period (2900–2373 B.C.) to the time of Hammurabi (1792–1750 B.C.). The interpretation of ‘Meluhha’ as ‘Indus’ is supported by evidence for trade in raw materials, common artifact styles and motifs among the two regions . In addition, contact among Mesopotamia and the Egyptians began prior to the Early Dynastic period in Egypt (3050–2686 B.C.).

Although leprosy existed in Europe by 400 B.C. [13] it did not become widespread throughout the urban centers of that continent until the Medieval period, a time of expanding trade networks [6]. We argue that if leprosy evolved in Africa in the Pleistocene [12], it is unlikely to have spread into Asia and become a serious health issue until the late Holocene, when South Asia and Northeast Africa were part of a larger regional trade network that stretched across the Arabian Sea. We argue that transmission of *M. leprae* between Asia and Africa is most likely in the third millennium B.C., when India had extensive, wide-ranging networks for movements of peoples, goods, and potentially infectious diseases. This is a more likely time for transmission of communicable diseases such as leprosy than the Late Pleistocene migrations proposed by Pinhasi et al. [8] and thus supports the interpretation of the genetic data proposed by Monot et al. [12].

Further research should be performed to determine the geographic origin of the disease using an integrated approach that examines paleopathology and ancient DNA. Paleopathological evidence for the disease should be examined in the skeletal collections belonging to Indus Age sites. Urban centers in the height of the Indus Age and post-urban sites occupied in the second millennium B.C. should be of particular interest. In addition, the skeletal material from Balathal and from Indus sites should be investigated for evidence of ancient DNA from the *Mycobacterium*. There could also be well-preserved molecular evidence in Egyptian skeletons from the second or third millennium B.C. Although the first skeletal evidence from Dakhleh Oasis places the disease in Egypt only after 400–250 B.C. [23], the Ebers papyrus has been interpreted as evidence of more ancient knowledge of the disease by 1550 B.C. [3]. Assuming that DNA from the *Mycobacterium* can be obtained from individual 1997-1, genetic comparison of the strain from Balathal and additional skeletal specimens may provide new insights into the origin of the disease if a relationship could be demonstrated with either the Type I or II strains previously identified [12]. Until the origin of leprosy is confirmed through additional research, the significance of this individual from Balathal is that it marks the earliest skeletal evidence for lepromatous leprosy, demonstrating its presence in a North Indian population during a time of substantial interaction among populations throughout Asia, the Middle East, and Africa.
